# Phase contrast reflectance confocal brain imaging at 1650 nm

**DOI:** 10.1117/1.JBO.29.2.026501

**Published:** 2024-02-27

**Authors:** Patrick Delafontaine Martel, Cong Zhang, Andreas A. Linninger, Frédéric Lesage

**Affiliations:** a Polytechnique Montreal, Department of Electrical Engineering, Montreal, Québec, Canada; b Montreal Heart Institute, Research Center, Montreal, Québec, Canada; c University of Illinois, Department of Biomedical Engineering and Neurosurgery, Chicago, Illinois, United States

**Keywords:** cortical microscopy, intrinsic imaging, phase contrast microscopy, reflectance confocal microscopy, NIR-II microscopy

## Abstract

**Significance:**

The imaging depth of microscopy techniques is limited by the ability of light to penetrate biological tissue. Recent research has addressed this limitation by combining a reflectance confocal microscope with the NIR-II (or shortwave infrared) spectrum. This approach offers significant imaging depth, is straightforward in design, and remains cost-effective. However, the imaging system, which relies on intrinsic signals, could benefit from adjustments in its optical design and post-processing methods to differentiate cortical cells, such as neurons and small blood vessels.

**Aim:**

We implemented a phase contrast detection scheme to a reflectance confocal microscope using NIR-II spectral range as illumination.

**Approach:**

We analyzed the features retrieved in the images while testing the imaging depth. Moreover, we introduce an acquisition method for distinguishing dynamic signals from the background, allowing the creation of vascular maps similar to those produced by optical coherence tomography.

**Results:**

The phase contrast implementation is successful to retrieve deep images in the cortex up to 800  μm using a cranial window. Vascular maps were retrieved at similar cortical depth and the possibility of combining multiple images can provide a vessel network.

**Conclusions:**

Phase contrast reflectance confocal microscopy can improve the outlining of cortical cell bodies. With the presented framework, angiograms can be retrieved from the dynamic signal in the biological tissue. Our work presents an optical implementation and analysis techniques from a former microscope design.

## 1 Introduction

In biological tissue, water absorption and associated O-H vibrational states lead to increased attenuation of light around 1450 nm.^1^ However, since the scattering of light is mostly inversely proportional to the wavelength, higher wavelengths (1600 to 1850 nm) have an increased effective attenuation length.^2^ With the advent of new NIR-II sources and low noise detection in the same spectral range, difficulties associated with imaging in this range have been reduced and therefore increase the interest in high wavelength imaging *in-vivo* microscopy. Some notable work led to demonstrations of three photon imaging,^3^ confocal fluorescence imaging via quantum dots excitation^4^^,^^5^ and development of new probes targeting this spectral range.^2^^,^^6^

In recent work, development of a long-wavelength reflectance confocal microscope has demonstrated good endogenous imaging capabilities when exploiting the NIR-II band.^7^ Polarization filtering was used to maximize signal to noise ratio (SNR) leading to good imaging depth in tissue despite using low excitation power. However, some ambiguity in the intrinsic signal may arise when comparing vascular structures to myelinated axons. Such ambivalence was resolved in the aforementioned work via combining with molecular imaging, such as third harmonic generation (THG).

There exists a huge body of work in ophthalmology imaging on the potential of phase contrast to help identify cellular interfaces. Building from the standard adaptive optics scanning laser opthalmoscopy (AOSLO) imaging setup, phase contrast annexed to an AOSLO system was introduced by Sulai et al.^8^ The lateral separation of the microscope’s point spread function enhanced the overall contrast and ability to detect micro-features of the system.^9^ A similar approach has not been investigated in the brain since. However, usage of NIR-II spectral range exhibits reduced scattering of light, which may facilitate the implementation of phase-contrast imaging and prove beneficial if applied to the reflectance confocal microscopy setup.

In the absence of a femtosecond source to generate THG, vascular angiography could benefit from a technique similar to speckle analysis in optical coherence tomography (OCT). Based on high-frequency temporal filtering of the signal, OCT is able to retrieve erythrocytes paths *in-vivo*.^10^ A similar approach to the NIR-II reflectance confocal microscope could help distinguish axons from blood vessels in cortical tissue.

In this study, we investigate whether the combination of phase contrast scheme with NIR-II reflectance confocal microscope can provide intrinsic contrast to cells, including erythrocytes in the lumen. The study will show that combining this imaging setup with high frequency temporal filtering proves an efficient framework to detect the micro-vascular network structure (or angioarchitecture) and differentiate dynamic elements with flow such as blood vessels from static ones in the cortex. Our report describes the imaging setup, methods for dynamic structures imaging and *in-vivo* test with mice’s skull left intact to test the capabilities of the custom microscope.

## 2 Methodology

### 2.1 Animal Groups and Surgery

The Animal Research Ethics Committee of the Montreal Heart Institute approved all procedures described here, in accordance with the Canadian Council on Animal Care recommendations and the protocol for this study was accepted under the ID 2023-3257. Mice underwent a diet of TEKLAD GlobaL 19% protein extruded rodent diet (Envigo) and were kept under a light/dark cycle of 12 h. Clean drinkable water was at all times available. We used n=9 C57BL/6 J mice (5M and 4F) kept in separate cages. Two distinct surgical procedures were used to enable cortical imaging with either intact skull or through a conventional craniotomy. For the craniotomy preparation, a cranial surgery was performed before the imaging sessions following a protocol identical to Lu et al.^11^ During the surgery, body temperature, respiration rate and heart rate were monitored (LabeoTech, Canada). Lidocaine was injected onto the surface of the scalp for analgesia and the scalp was removed all under anesthesia (3% isoflurane in oxygen). For the conventional cranial window, a micro drill was used to open the skull. A quarter wave plate (QWP, WPH502, Thorlabs, Inc.), cut in four square pieces, were used as coverslips. UV sensitive dental cement was used to fix the QWP and UV light was shone for 20 s to cure the dental cement while a custom arm kept the cranial window slightly pressured on the cortical surface. For intact skull imaging, refractive index matching acrylic glue (zap-a-gap CA+) was used to fix the reshaped (QWP) directly on the skull. Finally, a titanium bar was glued onto the head to serve as an anchor point to maintain the mouse brain fixed during imaging sessions. The imaging sessions were then performed while under isoflurane anesthesia (reduced to 1% to 2%), on the heating pad, while keeping the head of the mouse immobile throughout the imaging process (see Fig. S1 in the Supplementary Material for graphical representation). All experiments were terminal.

### 2.2 Optical System

**Fig. 1:**
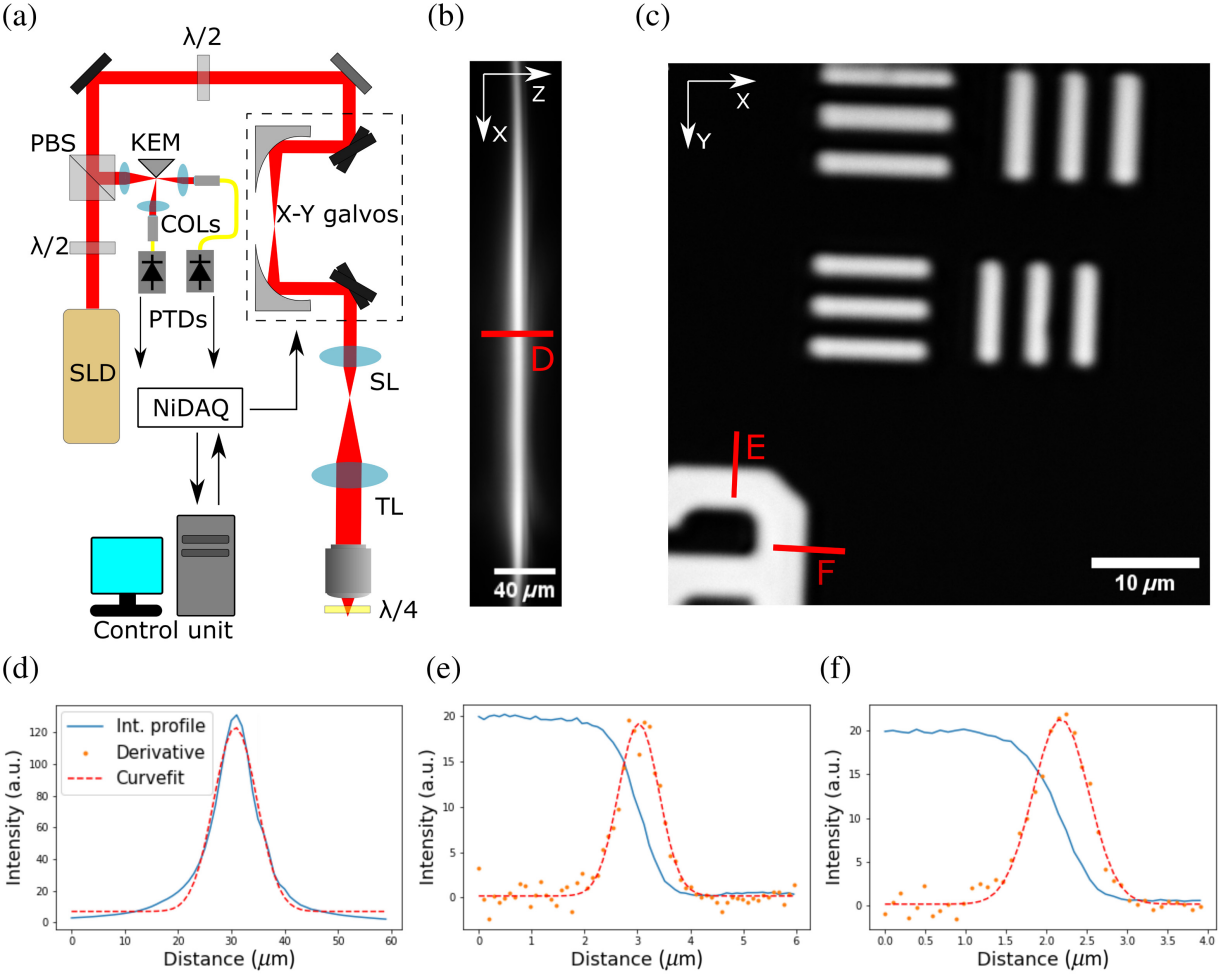
Schematic presentation of the reflectance confocal microscope designed in this work (a). Polarization separation is provided by the quarter-wave plate which feeds back reflected signal to descan the received light. The knife-edge mirror is used to create the phase contrast imaging scheme. NiDAQ, National Instrument Acquisition card; SLD, superluminescent diode; λ/2, half-wave plate; PBS, polarization beam splitter; SL, scan lens; TL, tube lens; and λ/4, quarter-wave plate. KEM, silver-coated knife-edge mirror and PTDs, photon detectors. Axial image stack with 1  μm steps has been acquired (b) providing an axial LSF. The horizontal plane of the image shown is the depth axis. A USAF 1951 resolution target has been imaged to characterize the lateral resolution (c) and the ESF method was chosen. The intensity profile in the axial direction and lateral direction are shown and the FWHM was used as a metric for resolution. (d)–(f) Each intensity profile is plotted. For the axial resolution (d), a Gaussian curve is plotted over the data to provide the FWHM. The vertical and horizontal edge spread function are also plotted (e, f). Retrieval of FWHM is performed through the derivative of the line spread function and curve fitting of a Gaussian profile.

The optical system design is similar to what is described in Xia et al.^10^ and is shown in Fig 1(a). A polarized superluminescent diode source (FPL1059P, Thorlabs) injects a 1650 nm light in the system with a collimator (ZC618SMA-C, Thorlabs). A first half-wave plate is used to optimize the transmitted light in a polarized beam splitter and a second half-wave plate changes the polarization of the excitation light to enable redirection of the collected light to the detectors through a polarization filtering scheme (see below). The raster scan setup is made from two galvanometric mirrors (Thorlabs) combined through two parabolic 90 deg off-axis mirrors (MPD129-M01, Thorlabs) to discard any beam walking effect. The excitation light is then expanded by a 6× beam expander to fill the back aperture at the objective plane. In order to dissociate the excitation light and the spurious reflections on the lenses from the collected light, a quarter-wave plate is inserted in the optical path after the microscope objective (XLPLN25XWMP2, Olympus) and serves as cranial window (see above). The back-and-forth passage in the later plate will cause a rotation of the polarized light, which will be refocused and descanned to be reflected at the polarization beam splitter. Moreover, a heavy water drop was used to couple the microscope objective to the cranial surface and to provide a liquid media, which has low water absorption at 1650 nm. A 60 mm lens is used to focus the detected light on the edge of the silver coated knife edge mirror (MRAK25-P01) and two other lenses are placed to collimate the separated beams back towards collimators (PAF2P-18C) fixed to fibers having a single-mode diameter of 10  μm. This configuration to enable phase contrast corresponds to a wave vector domain separation in two halves. Hence, reproducing the Fouceault effect in both beams sent to the fibered detectors. This decomposition of the detected beam in two parts similar to the positive and negative Zernicke polynomial $Z_{3}^{1}$ as shown to enhance the visibility of single cells in retina imaging.^12^^,^^13^ Fibers were connected to superconducting nanowire single-photon detectors (Opus One, Quantum Opus). This configuration provides a pinhole equivalent to ∼0.8 Airy unit. The galvanometric mirrors were operated via an acquisition card (Multifunction I/O device, National Instruments) connected to a computer. Both detector outputs were connected to counter channels and were synchronous with the raster scan setup. The signal is sent back to the computer to create the final image. Subtraction of both images for phase-contrast imaging is done via custom software implementation and the optical system was programmed in python.

### 2.3 Repeated Line Acquisition Scheme

The ability of reflection confocal microscopy to image cellular structures is an opportunity to take advantage of temporally changing signals from cortical tissue. Using a methodology inspired from the OCT angiography,^14^ speckle temporal analysis may be useful in this imaging system to provide similar outputs. Beyond standard scanning to generate images, we applied the repeated line method and acquired up to 100 images at random locations with a sampling frequency of ∼250  kHz per point to investigate the capabilities of the system at retrieving vascular structures.

## 3 Results

### 3.1 Optical System Characterization

wTo quantify the imaging capabilities of the optical system, the lateral edge spread function (ESF) and axial line spread functions (LSFs) were analyzed. These methods were preferred as the smallest element of the available USAF 1951 target was the 6’th element of the 7’th group, which are shown in the Fig. 1(c). For the ESF, a USAF 1951 resolution target (R1L1S1P, Thorlabs) was used to create a strong contrast with minimal power input (12.5 nW) to ensure no saturation at the photon counter detection rate. Mapping the ESF from the number near the 7’th group and 5’th element gives the lateral LSF via the derivative. An exponential curve fit provides a lateral full width half max (FWHM) of 920±10  nm in the horizontal direction and 820±10  nm in the vertical direction. This discrepancy between both axes may arise from the parabolic mirror alignment. However, the Rayleigh criterion for lateral resolution with our setup provides a value of ∼830  nm, which is in the same range of values as the LSF here analyzed, which tends to indicate that our setup is almost diffraction limited [Figs. 1(b) and 1(c) and 1(e) and 1(f)]. For the axial LSF, a mirror surface was imaged with a stack of 50 images separated by 1  μm steps. A curve fit over the resulting intensity profile shows an axial FWHM of 8  μm [Fig. 1(d)].

### 3.2 Phase Contrast Imaging can Distinguish Individual Cell Bodies

**Fig. 2:**
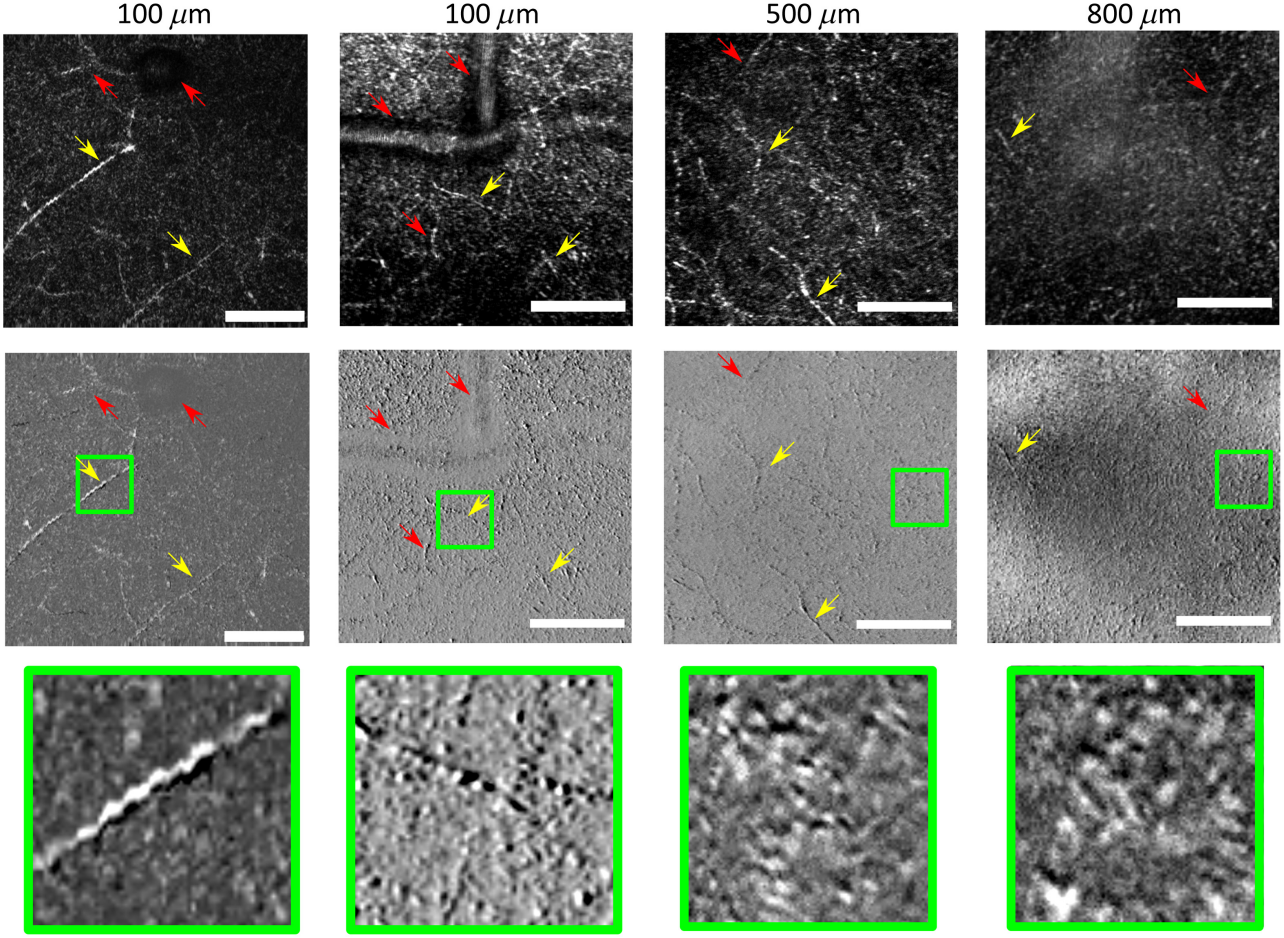
Imaging capabilities and phase contrast imaging at different depths. The images on the upper row are the addition of both channels of the reflectance confocal microscope providing the complete intrinsic signal from different cortical components. The subtraction of both channels, shown in the middle row, provides the phase contrast images which delineated the edges of the blood vessels (red arrows) and myelinated axons (yellow arrows). Close-ups of 30  μm field of view of cellular constituents are presented in the last row. Green boxes present the respective close-up images of cells.

The intrinsic contrast retrieved from confocal imaging at this wavelength is partly confounded since different morphological structures can exhibit relatively similar signals. To provide more insight on the cortical constituents, a phase-contrast scheme was introduced to the reflective confocal microscope. 150  μm wide images were acquired at different depths to test the system. To reproduce the intrinsic common signal from reflective confocal microscopy the signal of both collection channels can be added. Such acquisitions capture different structures such as myelinated axons, blood vessels and other glial cells up to 800  μm. As shown in Fig. 2, red and yellow arrows point to vascular structures and myelinated axons respectively. By subtraction of both channels, the phase contrast scheme delineates the reflective surface of the cellular bodies. Normalizing the signal background to a value around 0 outlines the cellular components in the image and clear out the low spatial frequency signal. Moreover, a left-right differentiation enhances cellular interfaces producing a high spatial frequency signal in the tissue. Anatomical features acquired in the cortex conform to descriptions found in atlases. However, the distinction between myelinated axons and small vessels remains hard to identify, since the only difference is the edge of each structure. A quantitative framework could be a useful tool to automatically label vasculature and anatomical features such as axons.

### 3.3 Temporal Analysis Outlines a Clear Vascular Network

From the images collected by the reflective confocal microscope, raw outputs showed that some tubular structures had internal intermittent signals. Hence, by performing a temporal variance computation ($\sigma_{t}$) of the differential image (Δf(x,y,t)) over the temporal mean ($\mu_{t}(x,y)$) of the addition of the two channels ($f_{1}(x,y,t)$, $f_{2}(x,y,t)$) over “T” images, dynamic structures were outline from static components in the image

$$\sigma_{t}\;=\frac{\sum_{t=1}^{T}(\Delta f(x,y,t)-\mu_{t}(x,y){)}^{2}}{\mu_{t}(x,y)},\;$$ (1)

where :

$$\Delta f(x,y,t)=f_{1}(x,y,t)-f_{2}(x,y,t),$$ (2)

and

$$\mu_{t}(x,y)=\frac{\sum_{t=1}^{T}(f_{1}(x,y,t)+f_{2}(x,y,t))}{T}.$$ (3)

**Fig. 3:**
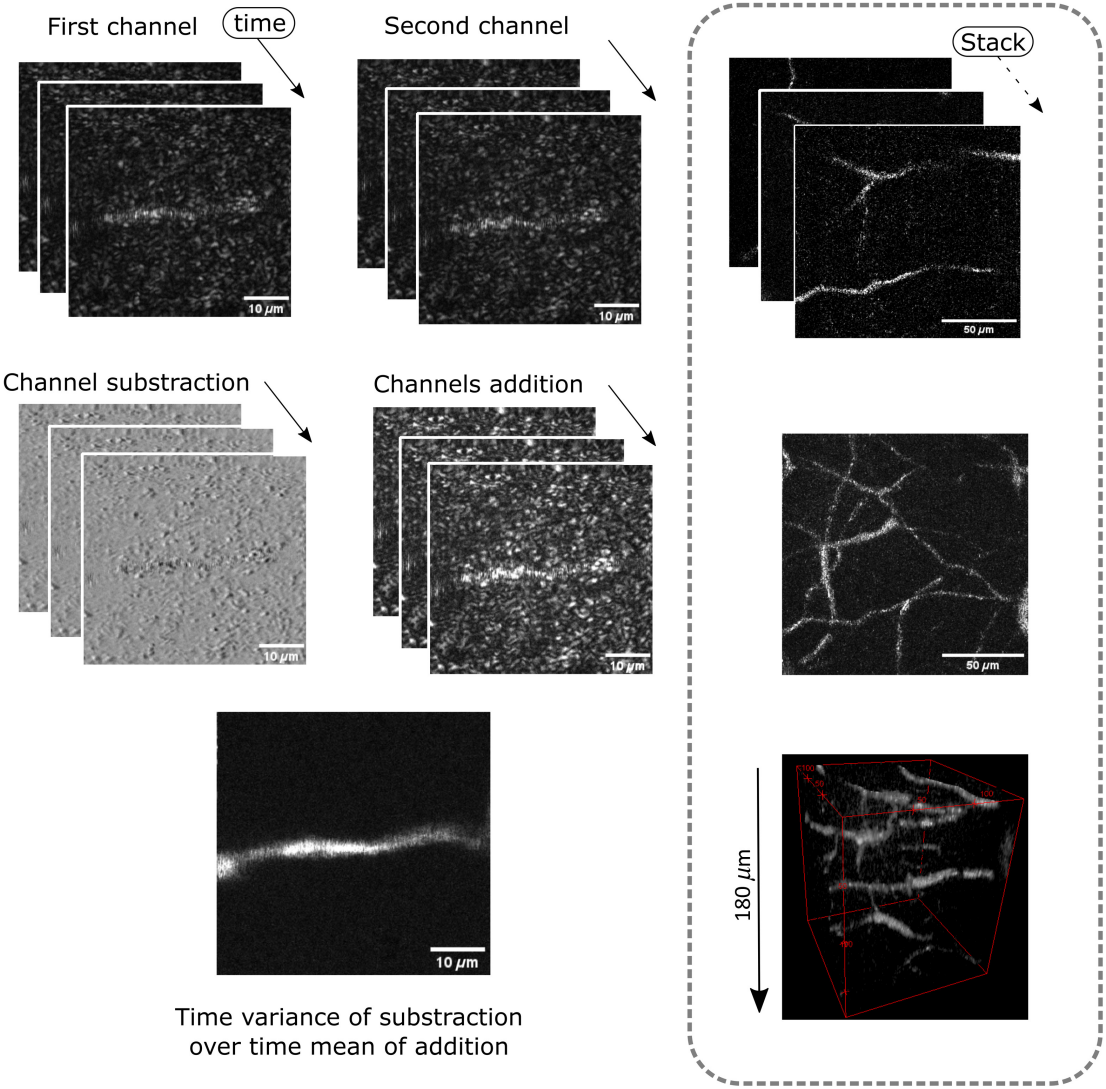
Raw images and signal processing routine from the reflective confocal microscope with a phase contrast scheme. The raw images from both channels are subtracted from one another to create the phase contrast image. Repeated acquisition on the fast axis scanner dimension provides temporal information which is retrieved via a temporal variance computation divided by the addition of the two raw images. The resulting image provides a vessel network map arising from the erythrocyte passage in the blood vessels. In the dotted box, the same image processing technique is repeated over 180  μm thickness in the somatosensory cortex to retrieve the volumetric vascular network.

To provide clear images of the vascular networks, a typical acquisition at 250  μm deep, 256 by 256 pixels at 102 kHz sampling per point with three consecutive line acquisition is shown at Fig. 3. The resulting image is the mean of five consecutive line scan acquisitions, which brings the time of acquisition to provide an image of the vascular component to around 10 s. Repeating this acquisition process while lowering the microscope objective down enables the digital reconstruction of angiograms. As shown in Fig. 3 in the dotted box, the same processing methodology is applied to an imaging sequence while 3  μm steps provided by a linear stage drive the microscope’s objective down towards the cortex. The image stack presented starts at 100  μm and goes up to 280  μm deep in the somatosensory cortex.

### 3.4 Vascular Components can be Retrieved up to a Depth of 800  μm

**Fig. 4:**
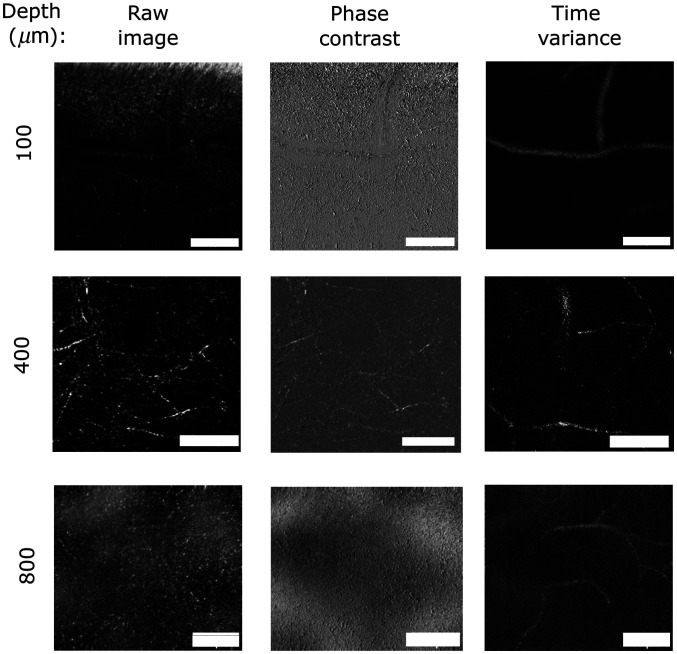
Demonstration of vascular component retrieval at different depths. For each depth, the raw signal, the subtraction of both channels, and the variance computation are presented. Tubular components in the raw signal can be attributed to either myelinated axons or blood vessels, but the temporal computation scheme allows to differentiate dynamic vascular structures from static elements, i.e., axons. All scale bars are 50  μm wide.

To highlight the potential for intrinsic imaging in the NIRII window, imaging sequences were performed to retrieve vascular structures up to 800  μm deep in the somatosensory cortex. Examples at different depths of raw signal from one channel, the presented phase contrast scheme and the temporarily changing structures highlighted by the variance computation are shown in Fig. 4. The acquisition time was increased by performing a higher number of repeated lines of acquisition in the image. For instance, at 400  μm, five consecutive repetitions for each line was performed. Deeper at 800  μm, 10 repetitions were acquired thus increasing the acquisition time at different depths accordingly. Trials to recover vascular signals over 800  μm were unfruitful even with 20 repetitive acquisitions.

### 3.5 Erythrocytes can be Monitored

**Fig. 5:**
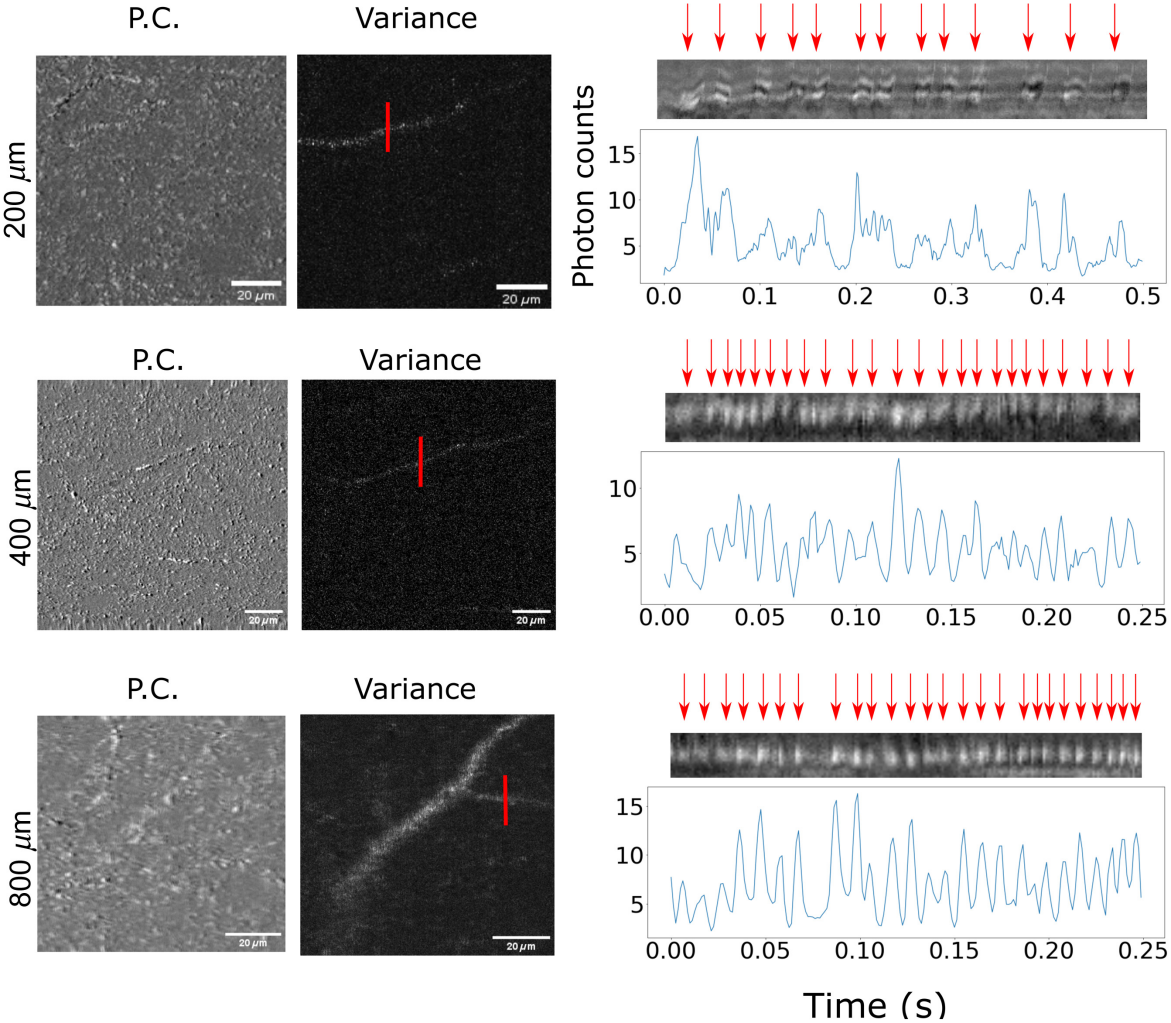
Demonstration of erythrocyte monitoring. The variance computation of the images can generate a vascular network map that incorporates small capillaries in the cortex. Using the fast axis scanning mirror at 800 hz and by placing the confocal microscope on top of the detected capillary enables the visualization of the erythrocytes passage in the lumen.

Rapid scans of dynamic structures were performed in order to observe the erythrocytes pass-by in capillaries. By fixing the scan length to 20  μm with a line rate of 800 Hz over the lumen of a capillary, erythrocyte passage was monitored. As shown in Fig. 5, this imaging mode can be performed up to 800  μm deep in the cortex. Comparison between different depths showed a similar level of signal coming from erythrocytes in the lumen when increasing the power of the excitation light.

### 3.6 Intact Skull Imaging can also Retrieve Cortical Cellular Structures

**Fig. 6:**
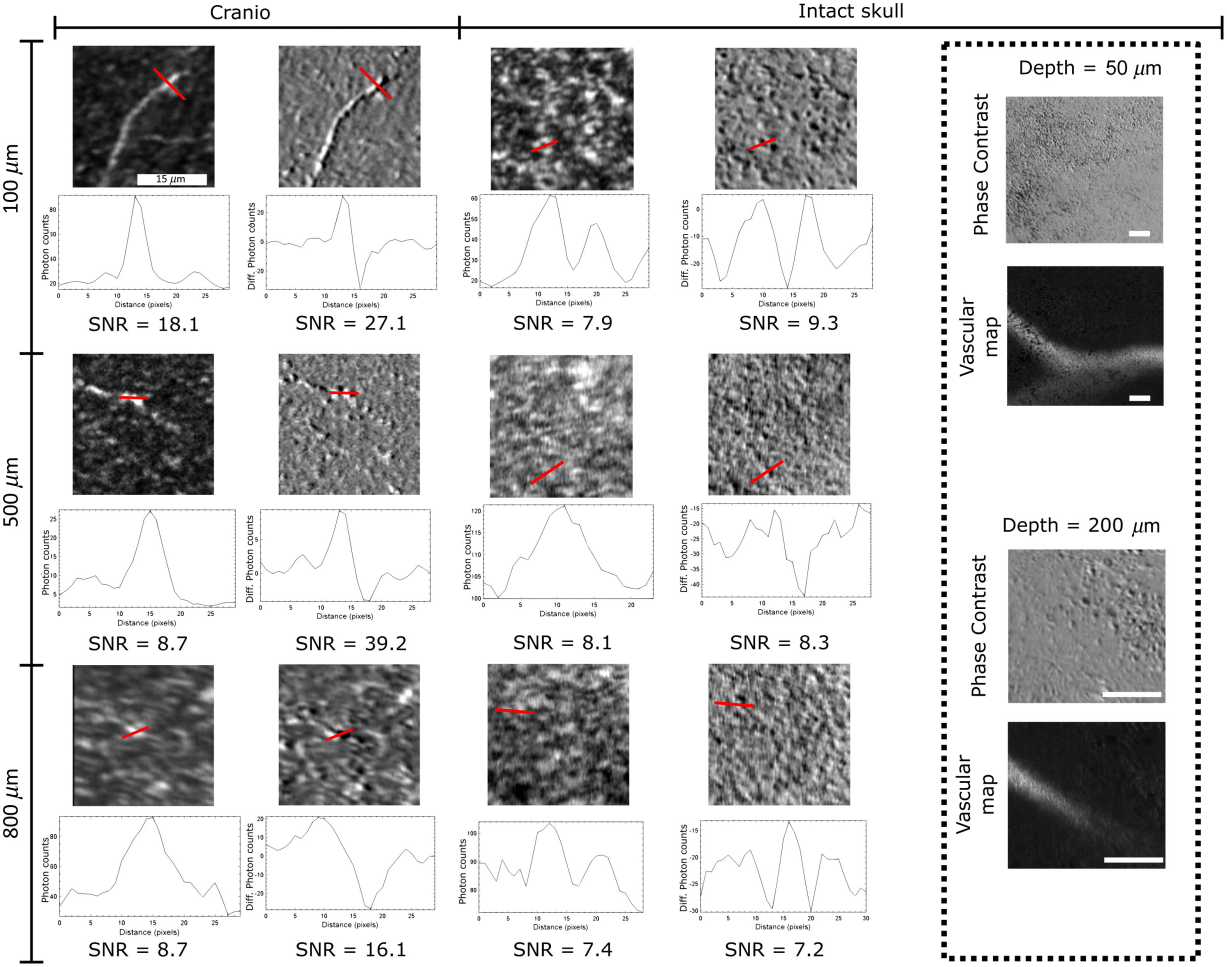
Comparison between imaging through intact skull and a cranial window. SNRs are indicated next to the images at different depths. Images through the intact skull exhibit lower SNRs than their counterpart at the same depth through a cranial window. Through the skull, cells bodies are distinguishable up to 500  μm but no vessels were retrieved past 200  μm using the temporal filtering technique. Scale bars at 15  μm.

Usage of NIR-II spectral range enables us to acquire micro-anatomical images through the skull with minimal scattering. Images at different depths through the skull were compared to acquisitions obtained after craniotomy. Figure 6 shows acquisitions at different depths of cells’ bodies comparing a complete craniotomy and an intact skull imaging with a 20  μm field of view. Up to 500  μm, the images reproduce glomerular bodies as observed in the craniotomy data. However, at 800  μm, the noise coming from the relatively high illumination hampered the nature of the signal and what appeared to be the cells body was lost in the noise. In contrast, during an open cranial window imaging, superior SNR provided a good delineation of the cellular constituents even at a depth of 800  μm. To quantify the effect, the SNR was computed as the square of the expected value divided by the standard deviation of the squared data. While the craniotomy images provide a decent SNR, the intact skull images exhibited lower SNR. When applying the vascular imaging protocol, only peripheral vessels up to 200  μm deep were able to be imaged with the temporal filtering method and no capillary vessels were retrieved in this manner either.

## 4 Discussion

### 4.1 Visible Structures from the Phase Contrast Reflectance Confocal Microscope

Reflectance confocal microscopy can provide convoluted signals with low selectivity for the biological constituents. This low selectivity is a result from the nature of the captured signal, which comes from reflected light onto the interface of media provided by changes in refractive index and absorption throughout the biological tissue. Past observations with phase contrast imaging focusing on lymphocytes through a transparent medium were able to depict the immune response from lipopolysaccharride induced inflammation.^12^ Such discerning power would be highly desirable for cortical imaging, which could eliminate the need for fluorescent dyes for cortical cells in some experiments. Using a molecular probe entails a biomolecular bond with the observed cell, which might interfere with their normal functions. The need for a biological probe is eliminated in phase-contrast acquisition, although the feasibility of such an intrinsic imaging technique in the presence of cortical tissue scattering needed to be investigated. Here, we demonstrated that the reduced scattering from the usage of the NIR-II spectral band could enable phase contrast up to 800  μm deep in the cortex. This result appears to be a considerable advance in the imaging depth of the technology since reflectance confocal imaging in the NIR range provides depth neighboring the 500  μm with similar craniotomy technique.^15^^,^^16^

Observing the images recorded with our microscope using phase contrast, the different anatomical structures, such as myelinated axons, blood vessels, and glial cells, seem to provide signals compatible with this mechanism and clearly delineate structures, such as seen in Fig. 2. Myelinated axons, distinguishable by their elongated shape and static signal, reflected a significant amount of light, probably arising from the myelinated sheets or Schwann cells primarily constituted of fatty molecules. Schwann cells may be seen as little bumps arranged in elongated columns. Depending on microscope placement and image resolution, these myelinated axons can be also seen as continuous tubules. In the cases of erythrocytes and glial cells, the change of refraction index at the cellular wall may be the mechanism reflecting the detected light. Since the phase contrast imaging is known to exhibit signals, which distinguish wavefront differences, the shadow observed in Fig. 5 is a good indicator of round surface projecting light back to the objective. Such detected light is then separated in two halves in the wave-vector domain via the split detection scheme, thus creating a sensitivity of the angle of incidence of light over the biological structure. In the literature of phase contrast imaging, most applications of epi-detection phase contrast scheme are used with a highly reflective surface beneath the sample in order to illuminate through the cells to shed light to internal structures.^17^ Since the usage of in-vivo models restrains the possibility of such methodology, signal coming from the cortical tissue is mainly the reflected light from the cell surface.

### 4.2 Repetitive Image Acquisition Detects a Vascular Network Topology

Temporal analysis of the reflective signal yields a vascular network in the imaging depth of the confocal microscope. This capability of revealing the path of dynamically flowing erythrocytes stems from their reflection in the vessels’ lumen. This variance imaging scheme was shown to delineate the vascular architecture with little signal contamination from static structures. The variance scheme while performing an image stack provided connectivity of descending vessels. This connectivity of the vascular map may arise from the erythrocyte brief passage through the imaging plane of the microscope, which is hard to decipher in the reflectance confocal microscope’s raw images. Variance imaging of axons aligned with the optical axis did not generate sufficient contrast because collinear orientation does not produce enough reflection, so that their signal appears static.

### 4.3 Phase Contrast Scheme Discerning Power Degrades with Depth

The phase contrast scheme helps to reduce the background signal that could come from the optics since common signals in both channels are subtracted to one another. Moreover, the polarization filtering of the spurious reflection is able to remove all the characteristic noise up to 10 nW under the objective. The very high sensitivity of the system is due to the use of superconducting nanowire single photon detector (SNSPD, Quantum Opus) photon counters. Even with this sensibility, no structure was visible past ∼1  mm of depth. A similar system has been shown to image over the visual cortex of mice up to a depth of 1.2 mm.^7^ The different cortical area and usage of a split detection scheme may be the cause that impaired the capabilities of deep light collection.

### 4.4 Imaging of Cortical Structures Through the Skull

Imaging through the intact skull deteriorated the overall sensitivity of the microscope to the reflective constituents of the cortical tissue up to 800  μm depth in the cortex. The signal collected provided vascular images only at superficial depths (under 200  μm), hence restricting the capabilities of the imaging setup to perform through skull longitudinal observation. It is clear that the deterioration of the resolution arises from the passage through the skull, which could be avoided with different techniques, such as skull optical clearing^18^ or adaptive imaging via frontwave manipulation.^19^ However, optical clearing requires access to the skull surface by fixing a QWP onto the surface of the skull, which prevents the manipulation required. Implementation of an adaptive optic component to the system may be more suitable for future experiments.

### 4.5 Limitations

While the proposed imaging system is relatively low cost and effective for generating phase contrast from intrinsic signals, the long imaging sequences needed for acquiring clear angiograms of the vascular structure seems to limit its potential. A way to reduce acquisition time could be via a fast scanning method with a resonant scanning mirror to push further the observation of biological cells’ functions in the cortical tissue.

On the methodology front, the usage of a QWP as a cranial window may be inconvenient regarding surgery manipulations and window clearance. Advent of high numerical aperture microscope objectives with integrated QWP would open the way to skull clearing method without the need for creating cranial windows.

Moreover, the QWP placement may be suboptimal since the waveplates used as cranial windows are optimized for collimated light. With the placement under the objective with a numerical aperture of 1.05, the angled incoming light may not rotate its polarization completely. Hence, this effect may reduce the intensity sent to the detectors. However, to minimize the background noise, the QWP after the microscope objective ensures that the spurious reflexions coming from the microscope objective lies in an orthogonal polarization from the detected signal, which is why the present configuration was preferred.

## 5 Conclusion

In this work, a phase contrast scheme was integrated in a long-wavelength reflectance confocal microscopy. Derived images enable the detection of the glomerular aspect of singular glial cells while rendering tubular structures, such as myelinated axons and vessels.

Moreover, by exploiting temporal variance, signals arising from the erythrocyte passage in the lumen of blood vessels enable the reconstruction of connected angiograms.

Intact skull imaging was also shown to be feasible, which could be useful in certain applications not requiring deep cortical imaging. Future work will aim to accelerate acquisitions and investigate contrast associated with recruitment of immune cells.

## Supplementary Material



## Data Availability

Images taken and post-processed in this work are available upon request.
