# Supplementary material for: Phase contrast reflectance confocal brain imaging at 1650 nm
Source: J Biomed Opt. 2024 Feb 27;29(2):026501. doi: 10.1117/1.JBO.29.2.026501 (PMC10898133; doi:10.1117/1.JBO.29.2.026501)
Supplement: Supplementary file 1 [file JBO_029_026501_SD001.pdf]

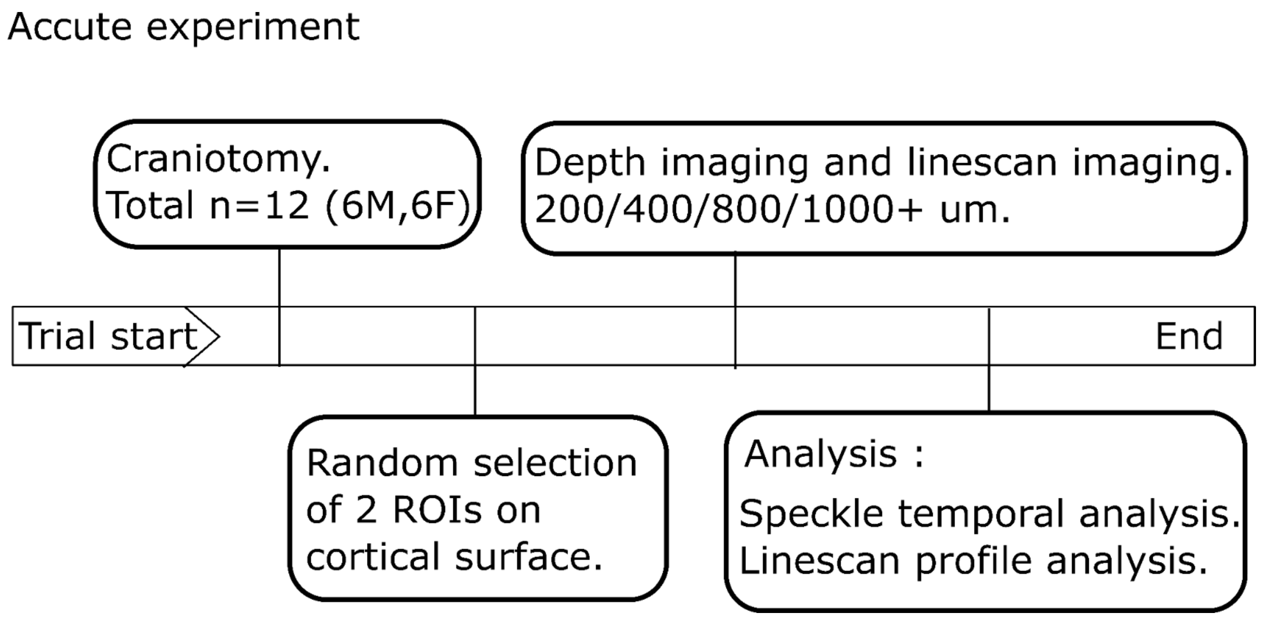

Supplemental Figure 1 : Graphical representation of the acute imaging sequence as described in the methodology.
